# Analysis of independent risk factors and construction of a predictive model for thyroid dysfunction in early pregnancy

**DOI:** 10.3389/fendo.2025.1631445

**Published:** 2025-11-14

**Authors:** Yiliminuer Keremu, Xiaolu Yu, Gang Zhao, Xu Chen, Liang Wang, Yan Zhang, Fan Guo, Xiumin Ma

**Affiliations:** 1 Department of Medical Laboratory Center, Tumor Hospital Affiliated to Xinjiang Medical University, Xinjiang Key Laboratory of Molecular Biology for Endemic Diseases, Urumqi, Xinjiang, China; 2 Department of Blood Transfusion, Affiliated Traditional Chinese Medicine Hospital of Xinjiang Medical University, Urumqi, Xinjiang, China; 3 Department of Laboratory, Kuitun Hospital of Ili Kazak Autonomous Prefecture, Kuitun, Xinjiang, China; 4 Department of Laboratory, The Fifth Affiliated Hospital of Xinjiang Medical University, Urumqi, Xinjiang, China; 5 Department of Laboratory, Bayinguoleng Mongol Autonomous Prefecture People’s Hospital, Korla, Xinjiang, China

**Keywords:** thyroid dysfunction, risk factors, prediction model, early pregnancy women, ROC curve

## Abstract

**Introduction:**

Thyroid dysfunction during early pregnancy significantly impacts maternal and fetal health, with risks including preeclampsia, preterm birth, and developmental abnormalities. This study aims to identify independent risk factors and develop a predictive model to enable early diagnosis and intervention, improving pregnancy outcomes through tailored clinical management strategies.

**Methods:**

We retrospectively analyzed the general information and relevant laboratory indicators of 2151 women in early pregnancy admitted to three Xinjiang hospitals from April 2021 to November 2024. The patients were divided into a normal thyroid function group (n=1490) and a thyroid dysfunction group (n=661). The test results were analyzed to screen for independent risk factors and constructed a predictive model.

**Results:**

Key findings revealed a 30.73% thyroid dysfunction incidence, including subclinical hypothyroidism (76.40%), hypothyroidism (12.86%), hyperthyroidism (6.35%), and subclinical hyperthyroidism (4.39%). Regional reference ranges were established as TSH (0.22–2.40) mIU/L and FT4 (13.54–20.26) pmol/L. Univariate analysis identified significant differences in A-TPO, A-TG, TSH, and FT3 (*P* < 0.05). Multivariate analysis confirmed A-TPO, TSH and FT3 as independent risk factors. The prediction model demonstrated excellent performance with an AUC of 0.911 (95% CI: 0.891-0.932), 0.874 sensitivity, and 0.955 specificity.

**Conclusion:**

The study demonstrated that A-TPO, TSH and FT3 were independent risk factors for thyroid dysfunction in women during early pregnancy. A predictive model was constructed based on these three indicators. Validation of the model’s performance indicates that it has good predictive capabilities.

## 1 Introduction

The thyroid gland, an essential endocrine gland located in the neck, is crucial for its secretion of hormones that significantly influence the body’s growth, development, and metabolic regulation (1). These hormones act on various systems throughout the body, playing a vital physiological role (2). However, pregnant women often suffer from thyroid disorders including clinical hypothyroidism, subclinical hypothyroidism, clinical hyperthyroidism and subclinical hyperthyroidism. These conditions can adversely affect maternal and infant health outcomes, posing a threat to their well-being (3, 4).

Women of childbearing age have a higher incidence rate of thyroid diseases in clinical practice. Studies have demonstrated that thyroid dysfunction in pregnant women can significantly affect fetal health (5). These effects not only involve fetal growth and development, but may also affect fetal brain development and neurological health (6, 7).

Currently, researchers worldwide have conducted extensive studies on thyroid dysfunction during pregnancy, accumulating substantial epidemiological data and identifying numerous potential risk factors such as advanced maternal age, positivity for thyroid autoantibodies (A-TPO, A-TG), abnormal body mass index (BMI), iodine nutritional status, and family history of thyroid disease (8–10). These studies have laid a foundation for understanding the distribution patterns and etiology of the condition. However, significant limitations remain in the existing research. Firstly, most studies have focused on cross-sectional prevalence surveys or analyses of single-factor associations, failing to integrate multiple risk factors. This makes it difficult to assess the independent effects and relative contributions of each factor after adjusting for confounders. Secondly, many studies rely on data from a single medical center, which limits the representativeness of their samples and the generalizability of their conclusions. Most critically, although a few studies have attempted to develop predictive models, they often lack rigorous and sufficient internal and external validation, particularly validation on multi-center datasets. This casts doubt on the clinical validity and reliability of such models, hindering their translation into practical clinical risk assessment tools.

To address these research gaps, this study aims to systematically collect data from a multi-center dataset involving several hospitals, including thyroid function indicators (FT3, FT4, TSH), autoantibodies (A-TPO, A-TG), and age in women during early pregnancy. Through univariate and multivariate logistic regression analyses, we will identify independent risk factors and further develop a comprehensive, individualized risk prediction model.

## 2 Materials and methods

### 2.1 Research object

The study included a total of 2151 pregnant women admitted to Tumor Hospital Affiliated to Xinjiang Medical University, the Fifth Affiliated Hospital of Xinjiang Medical University, and Xinjiang Bayinguoleng Mongolian Autonomous Prefecture People's Hospital between April 2021 and November 2024. All pregnant women were in the first 12 weeks of pregnancy. Participants had been divided into two groups based on their thyroid function: a normal thyroid function group and a thyroid dysfunction group. The normal thyroid function group consisted of 1490 women, while the thyroid dysfunction group had 661 women. The age range of the participants was 18 to 44 years, with an average age of 29.73 ± 4.11 years. All participants signed the informed consent form. This study protocol was approved by Ethics Committee of the Affiliated Tumor Hospital of Xinjiang Medical University (Approval No. K-2025141).

We collected and analyzed general information and relevant laboratory data from research objects. All pregnant women in the study did not experience any complications during pregnancy, and had no history of genetics, smoking, or alcohol consumption. The study also excluded participants with autoimmune deficiency, blood system diseases, pathological obesity, a history of hypertension or diabetes, and drugs that affect thyroid function.

We assessed data completeness and found no missing values for any of the analytical variables, including thyroid function indicators (A-TPO, A-TG, TSH, FT4, FT3) and relevant clinical data. Consequently, the final analysis included all 2151 participants based on complete data. Given the multicentric nature of the data, a generalized linear mixed model with ‘study center’ as a random intercept was employed to account for potential inter-center variation. All continuous predictors were standardized (z-scores) to improve model convergence.

### 2.2 Research methods and reagents

All study subjects were collected 5mL of peripheral venous blood, which was centrifuged to obtain serum for testing, and the samples were tested on the same day. The levels of free triiodothyronine (FT3), free thyroxine (FT4), thyroid-stimulating hormone (TSH), thyroid peroxidase antibodies (A-TPO), and thyroglobulin antibodies (A-TG) were measured using a Roche cobas601 electrochemiluminescence analyzer with the corresponding reagent kit.

### 2.3 Diagnostic criteria

The following reference ranges were used for the assessment of thyroid function: Reference Ranges: TSH: 0.09–2.50 mIU/L; FT4: 13.15–20.78 pmol/L.

Thyroid dysfunction was defined as follows: Hyperthyroidism: TSH< 0.09 mIU/L and FT4 > 20.78 pmol/L; Subclinical Hyperthyroidism: TSH< 0.09 mIU/L and FT4 within normal range; Hypothyroidism: TSH > 2.50 mIU/L and FT4< 13.15 pmol/L; Subclinical Hypothyroidism: TSH > 2.50 mIU/L and FT4 within normal range.

### 2.4 Statistical methods

The collected data were processed using SPSS27.0 software for simple correlation. Quantitative data with a skewed distribution were presented as the median and interquartile range, *P* < 0.05 was considered statistically significant. Multiple logistic regression analysis was performed on variables that were statistically significant, ultimately summarizing the independent risk factors associated with thyroid dysfunction in early pregnant women. Then a regression equation was established to predict the probability of thyroid dysfunction in this population. The reference interval was established using the nonparametric method (P_2.5_ – P_97.5_) as recommended by the CLSI C28-A3 guideline.

## 3 Results

### 3.1 The proportion of normal and abnormal thyroid function in early pregnancy women

Among 2151 pregnant women, a total of 661 cases (30.73%, 661/2151) were screened for thyroid dysfunction. There were 1490 healthy pregnant women, accounting for 69.27% (1490/2151). Within the group with thyroid dysfunction, the distribution was as follows: 85 cases (12.86%) with hypothyroidism, 505 cases (76.40%) with subclinical hypothyroidism, 42 cases (6.35%) with hyperthyroidism, and 29 cases (4.39%) with subclinical hyperthyroidism. The incidence of hypothyroidism was higher in women with thyroid dysfunction in early pregnancy, as shown in **Table 1**.

**Table 1:** The proportion of normal and abnormal thyroid function in women during early pregnancy.

Grouping	n (%)
Euthyroidism	1490 (69.27%)
Thyroid Dysfunction	661 (30.73%)
Hypothyroidism	85 (12.86%)
Subclinical hypothyroidism	505 (76.40%)
Hyperthyroidism	42 (6.35%)
Subclinical hyperthyroidism	29 (4.39%)

### 3.2 Establishment of reference values for TSH and FT4 indicators in early pregnancy women

Based on the calculation of the reference interval (P_2.5_~P_97.5_), the reference values for TSH (0.22~2.40) mIU/L and FT4 (13.54~20.26) pmol/L obtained in this study did not significantly differ from the recommended values of TSH (0.09~2.50) mIU/L and FT4 (13.15~20.78) pmol/L specified in the *Guidelines for the diagnosis and management of thyroid diseases during pregnancy and postpartum (2nd edition)* (11).

### 3.3 Comparison of baseline clinical characteristics between the two groups

This study included a total of 2151 women in early pregnancy, of whom 1490 had normal thyroid function (euthyroidism group) and 661 had abnormal thyroid function (thyroid dysfunction group). A comparison of the baseline clinical characteristics between the two groups is presented in **Table 2**. The results demonstrated significant differences (*P* < 0.05) between the thyroid dysfunction group and the euthyroidism group in terms of A-TPO, A-TG, TSH, FT4, and FT3. Specifically, the thyroid dysfunction group exhibited significantly higher levels of A-TPO, A-TG, and TSH, whereas FT4 and FT3 levels were significantly lower compared to the normal thyroid function group.

**Table 2:** Comparison of baseline clinical characteristics between the two groups.

Parameter	Euthyroidism (n=1490)	Thyroid dysfunction (n=661)	Z	*P*
age	29 (27~32)	30 (27~33)	-1.446	0.148
A-TPO	10.97 (6.60~16.04)	12.93 (7.41~23.62)	-5.596	<0.001
A-TG	15.99 (12.90~20.59)	17.50 (13.75~72.58)	-5.835	<0.001
TSH	1.35 (0.88~1.88)	3.22 (2.72~4.12)	-29.094	<0.001
FT4	16.67 (15.39~17.99)	15.95 (14.45~17.62)	-7.008	<0.001
FT3	5.12 (4.77~5.52)	5.03 (4.53~5.51)	-2.974	<0.001

### 3.4 Univariate logistic analysis of risk factors associated with thyroid dysfunction in early pregnancy

Univariate logistic regression analysis was performed on all candidate variables. The results revealed that A-TPO, A-TG, TSH, and FT3 were significant influencing factors for abnormal thyroid function (*P* < 0.05). Specifically, the odds ratio (OR) for TSH was 7.077 (95% CI: 5.949–8.419, *P<* 0.001), indicating that for every 1 mIU/L increase in TSH, the risk of abnormal thyroid function increased approximately 7 fold. Both A-TPO (OR = 1.004, 95% CI: 1.003–1.005, *P* < 0.001) and A-TG (OR = 1.001, 95% CI: 1.000–1.001, *P<* 0.001) also showed significant positive correlations, although their effect sizes were small. A weak association was observed between FT3 and abnormal thyroid function (OR = 1.060, 95% CI: 1.002–1.121, *P* = 0.043), while no statistically significant differences were found for age (*P* = 0.174) or FT4 (*P* = 0.072), as shown in **Table 3**.

**Table 3:** The results of the univariate logistic regression analysis of thyroid function.

Variables	β	SE	Wald	OR	OR (95%CI)	*P*
age	0.015	0.011	1.844	1.016	0.993–1.038	0.174
A-TPO	0.004	0.001	51.905	1.004	1.003–1.005	<0.001
A-TG	0.001	0.000	18.394	1.001	1.000–1.001	<0.001
TSH	1.957	0.089	487.650	7.077	5.949–8.419	<0.001
FT4	-0.026	0.015	3.229	0.974	0.946–1.002	0.072
FT3	0.058	0.029	4.097	1.060	1.002–1.121	0.043

### 3.5 Multivariate logistic regression analysis of risk factors associated with thyroid dysfunction in early pregnancy

To further identify independent risk factors for abnormal thyroid function in early pregnancy, variables with statistical significance from the univariate analysis were included in a multivariate logistic regression model. The results demonstrated that TSH and FT3 were independent risk factors for abnormal thyroid function in early pregnancy (*P* < 0.001). Among these, TSH exhibited the strongest association, with an adjusted odds ratio (aOR) of 9.840 (95% CI: 8.005–12.096). This indicates that for every 1 mIU/L increase in TSH, the risk of developing abnormal thyroid function increases by 9.84 times. The aOR for FT3 was 2.014 (95% CI: 1.704–2.382), meaning that for every 1 pmol/L increase in FT3, the risk of abnormal thyroid function is multiplied by 2.01. Although A-TPO also reached statistical significance (aOR = 1.002, 95% CI: 1.000–1.003, *P* = 0.045), its clinical relevance is likely limited due to the very small effect size. In contrast, A-TG did not show an independent association (*P* > 0.05), as shown in **Table 4**.

**Table 4:** The results of the multivariate logistic regression analysis of thyroid function.

Variables	β	SE	Wald	OR	OR (95%CI)	*P*
A-TPO	0.002	0.001	4.007	1.002	1.000–1.003	0.045
A-TG	0.000	0.000	0.720	1.000	1.000–1.001	0.396
TSH	2.286	0.105	471.423	9.840	8.005–12.096	<0.001
FT3	0.700	0.085	67.222	2.014	1.704–2.382	<0.001

### 3.6 Establishment of predictive model and ROC curve analysis

Based on the results of the multivariate logistic regression analysis, three independent risk factors are identified: A-TPO, TSH and FT3. These factors are used to establish a regression equation with their corresponding regression coefficients (β):


$$\begin{array}{c} \text{Logit}(\text{P})\;=\;-9.434\;+\;0.002\;\times \;\text{A}-\text{TPO}\;+\;2.286\;\times \;\text{TSH}\\ +\;0.700\;\times \;\text{FT}3 \end{array}$$

The predicted probability of abnormal thyroid function in women during early pregnancy is denoted as P. It is calculated using the following equation:


$$\text{P}\;=\;\frac{1}{1+{\text{e}}^{-\text{Logit}(\text{p})}}\;$$

**Figure 1:**
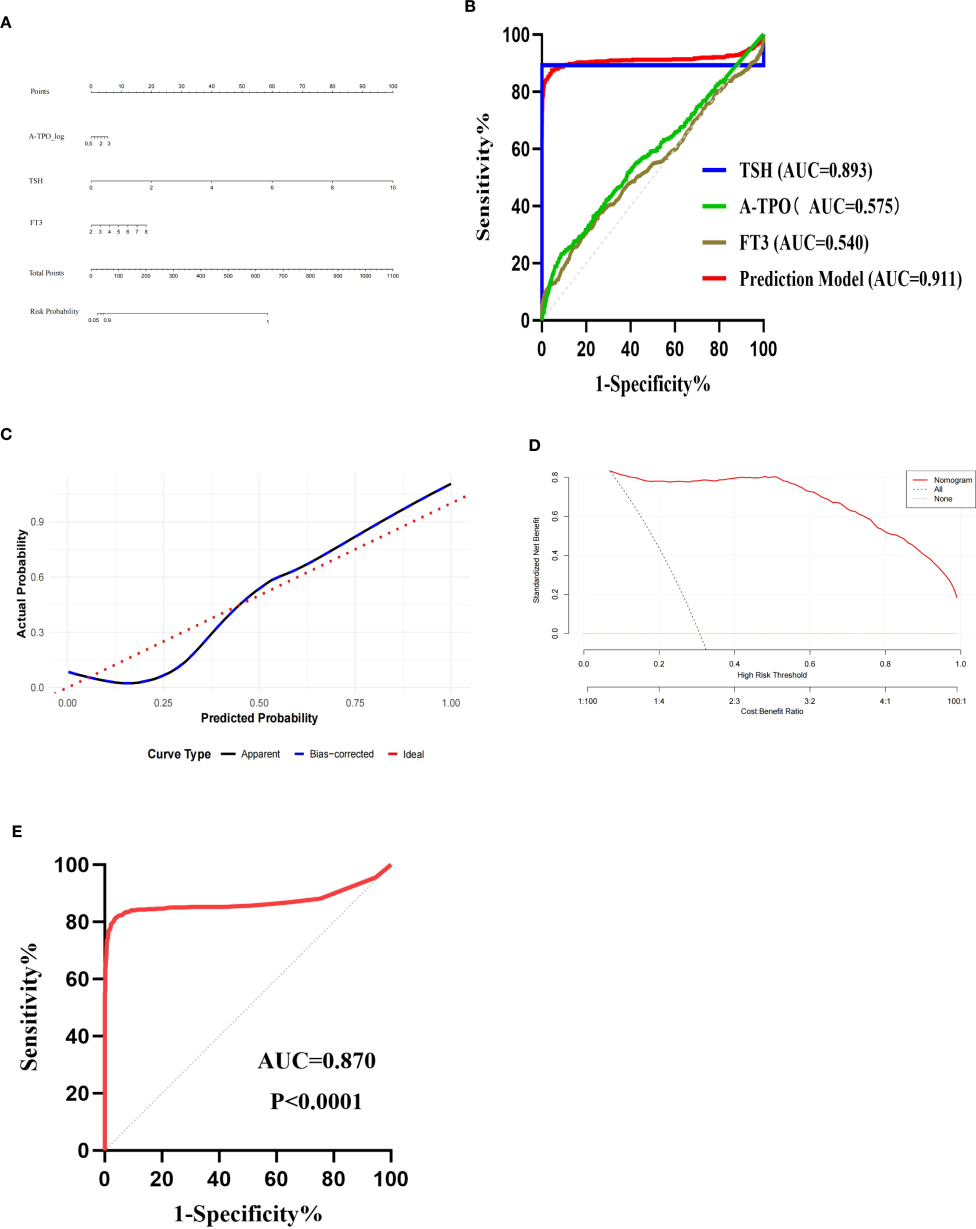
Validation of predictive model. **(A)** Nomogram of the predictive model for thyroid dysfunction in women during early pregnancy; **(B)** ROC curves of the prediction model and each independent risk factor; **(C)** Calibration curve; **(D)** Decision curve analysis (DCA) curve; **(E)** ROC curve of validation set.

A nomogram is constructed using R software, as shown in **Figure 1A**. The analysis reveals that the area under the receiver operating characteristic curve (AUC) of the predictive model is 0.911 (95%CI: 0.891–0.932), with a Youden index of 0.829, a cut-off value of 0.416, sensitivity of 0.874, and specificity of 0.955, as illustrated in **Figure 1B**. This indicates that the model has better discriminative ability compared to the individual prediction effects of A-TPO, TSH and FT3 alone. The predictors in the final model were evaluated for multicollinearity. As demonstrated by variance inflation factor (VIF) values all being less than 2, no significant multicollinearity was present (refer to **Supplementary Table S1** for detailed results).

### 3.7 Validation of predictive model

The Bootstrap method is used for model self-validation. Calibration analysis on the independent validation set (n=2151) demonstrated the model’s excellent calibration performance. The model achieved a Brier score (mean squared error) of 0.00807, a mean absolute error of 0.076, and a 90th percentile absolute error of 0.159, indicating that the predicted probabilities were in close agreement with the observed frequencies. Both the calibration curve and the self-validation using the Bootstrap method demonstrate that the model has good predictive accuracy and consistency, indicating that the predicted risks align well with the actual occurrence risks, as shown in **Figures 1C, D**.

This study conducted external validation of the model using 1619 women in early pregnancy who visited Kuitun Hospital in Ili Kazakh Autonomous Prefecture from January 2021 to December 2024. The AUC of the validation set was 0.870 (95% CI: 0.843–0.896), with a sensitivity of 0.816 and a specificity of 0.961, which was comparable to the performance of the training set (AUC = 0.911), as shown in **Figure 1E**. In the independent validation set (n=1619), the model demonstrated a calibration slope of 0.768 (95% CI: 0.672-0.891) and an intercept of 0.201 (95% CI: 0.080-0.335). These values respectively indicate a tendency for overfitting and a slight overall underestimation of risk. In the future, it is necessary to expand the sample size to verify its applicability among women in early pregnancy in more regions of Xinjiang.

The mixed-model analysis indicated that the variation in baseline risk across study centers was not statistically significant (variance of random intercept = 13.18, *P* = 0.632), suggesting that center effects did not substantially influence the primary outcomes. The fixed effects estimates, adjusted for center, are reported below (refer to **Supplementary Table S2** for detailed results).

## 4 Discussion

Through this multicenter study, we established the first specific reference intervals for TSH and FT4 in early pregnant women from the Xinjiang region, laying the groundwork for precise diagnosis in this population. Building upon these localized criteria, we further developed and validated a practical clinical prediction model. By integrating three core biomarkers—A-TPO, TSH, and FT3—this model reliably identifies high-risk individuals for thyroid dysfunction, a performance that was confirmed through rigorous internal and external validation. Consequently, our work provides a comprehensive solution, spanning from diagnostic standards to risk screening.

Since the 1980s, Dutch scholars detected thyroid hormones in the umbilical cord blood of neonates with congenital hypothyroidism and those with thyroid hormone synthesis disorders (12). This discovery first demonstrated that maternal thyroid hormones can be transferred to the fetus through the placenta. Later, American scholars published the clinical research titled “*Maternal thyroid deficiency during pregnancy and subsequent neuropsychological development of the child*”, which further supported the findings of Dutch scholars and emphasized the important role of maternal thyroid function in fetal brain development (13). Since then, the relationship between thyroid diseases in pregnant women and fetal development has become a research hotspot in obstetrics.

Clinical applications have shown that the reference ranges for TSH, FT4, and TT4 during pregnancy are different from those of the general population. *The 2017 American Thyroid Association (ATA) guidelines for the diagnosis and management of thyroid disease during pregnancy and the postpartum period (*14) stated that it was recommended for regions and institutions to establish pregnancy-specific serum thyroid function reference ranges. If pregnancy-specific thyroid function reference ranges were not available, the ATA guidelines suggested using 4.0 mU/L as the upper limit of normal for TSH in the first trimester of pregnancy. This study established the reference ranges for TSH and FT4 in women during early pregnancy in Xinjiang based on the collected data. The result showed that the reference range for TSH was (0.22-2.40) mIU/L and for FT4 was (13.54-20.26) pmol/L, which were not significantly different from the recommended reference ranges in the *Guidelines for the diagnosis and management of thyroid diseases during pregnancy and postpartum (2nd edition) (*11). This study referred to the diagnostic criteria in the Interpretation of the “*Guidelines for the Diagnosis and Management of Thyroid Diseases During Pregnancy and Postpartum (2nd Edition)*” *(*15) published in 2020. The results showed that among the 2151 pregnant women, 69.27% (n=1490) have normal thyroid function, while the incidence of thyroid dysfunction is 30.73% (n=661).

The biochemical indicators commonly use in clinical practice to assess thyroid function (16), collectively known as the full thyroid function panel, mainly include serum triiodothyronine (T3), thyroxine (T4), free T3 (FT3), free T4 (FT4), thyroid-stimulating hormone (TSH), calcitonin (CT), thyroglobulin (TG-II), as well as antibodies such as thyroid peroxidase antibody (A-TPO), thyroid-stimulating hormone receptor antibody (TRAb), and thyroglobulin antibody (A-TG). Pregnancy has a significant physiological impact on the thyroid gland and its metabolic functions. Hormonal changes and increased metabolic demands during pregnancy may lead to alterations in the biochemical indicators of thyroid function. Thyroid dysfunction in pregnant women can lead to adverse pregnancy outcomes such as gestational diabetes and fetal growth restriction (17, 18). This study retrospectively analyzed the age and relevant serological examination indicators of women in early pregnancy. Through univariate and multivariate logistic regression analysis, TSH, FT4, and FT3 were identified as independent risk factors for thyroid dysfunction in women during early pregnancy. Based on these factors, a predictive model was constructed.

The selection of A-TPO, TSH, and FT3 as the core predictors in our model is strongly supported by their distinct and complementary roles in thyroid pathophysiology. Positivity for A-TPO is a well-established marker of underlying autoimmune thyroiditis, representing the most common etiology for thyroid dysfunction (19). An elevated TSH level serves as the primary and most direct signal from the pituitary gland indicating reduced thyroid hormone output, thus identifying overt hypothyroidism (20). The inclusion of FT3, the biologically most active thyroid hormone, is a particular strength of our model. Its levels are increasingly recognized as a sensitive indicator of peripheral thyroid hormone status, potentially offering a more dynamic reflection of metabolic demand during early pregnancy than FT4 alone (21). From a clinical perspective, the practical utility of this model is significant. The derived risk score—which could be operationalized as a simple chart or an online calculator—provides clinicians with an immediate and objective tool to rapidly stratify early pregnant women. This facilitates a precision case-finding strategy, which strikes a crucial balance between the impracticality and cost of universal screening and the documented inadequacy of traditional risk-factor-based approaches, which miss a substantial proportion of affected women. The fact that our model maintained its performance across a multicenter dataset strongly suggests its potential for broad applicability in diverse clinical settings, moving it beyond a mere statistical exercise toward genuine clinical utility. The ROC curve analysis in this study showed that the AUC was 0.911 (95%CI: 0.891-0.932, *P* < 0.0001), with a sensitivity of 0.874 and a specificity of 0.955, indicating that the model had strong diagnostic ability. The calibration curve demonstrated that the model performed well in clinical applications. The established prediction model has good predictive ability for thyroid dysfunction in women during early pregnancy, and the evaluation indicators in the model are relatively easy to obtain in clinical practice. It has good clinical application value in identifying thyroid dysfunction in women during early pregnancy.

The innovation of this study lies in the construction of a risk prediction model for thyroid dysfunction in women during early pregnancy in the form of a nomogram, forming a scoring system with specific numerical values, which provides convenience for clinical practice. While this study offers valuable insights, it has several limitations that warrant attention. First, although the case-control design allowed for efficient initial model development, it inherently limits the ability to establish causal timelines—a strength of prospective cohort studies. Second, despite the benefits of a multicenter design, all participants were from Xinjiang, which may limit the generalizability of our findings to populations with different genetic backgrounds or iodine nutritional statuses. Third, as with all clinical studies relying on biomarker measurements, variations in laboratory techniques and assay kits across centers may have influenced the absolute values of thyroid parameters. Due to the retrospective nature of this study, we were unable to obtain complete clinical data for all patients, such as BMI, iodine nutritional status, and HCG levels. Consequently, this has limited the scope of predictor variables that could be included in our model. Finally, the model demonstrated some calibration error in the external validation set, possibly due to spectrum effects or case-mix differences between the training and validation cohorts. Despite imperfect calibration, the model’s strong discriminative ability (AUC = 0.911) supports its clinical utility in distinguishing between high- and low-risk patients.

Future research should focus on the following key directions: The priority is to validate our model’s efficacy through prospective intervention studies to determine whether screening and subsequent management based on this risk score can ultimately improve maternal and infant health outcomes. Furthermore, external validation in more geographically and ethnically diverse populations is essential to confirm its general applicability. Finally, integrating additional baseline clinical data and novel biomarkers could further enhance the model’s predictive accuracy and clinical relevance.

## 5 Conclusions

Our study revealed significant differences in the levels of A-TPO, A-TG, TSH, FT4, and FT3 between euthyroid and thyroid dysfunction groups among women in early pregnancy (*P* < 0.05). It proposed reference ranges for TSH (0.22-2.40 mIU/L) and FT4 (13.54-20.26 pmol/L) in women during early pregnancy in Xinjiang region. Three independent risk factors were identified through univariate and multivariate logistic regression analyses. By establishing a regression prediction model based on A-TPO, TSH and FT3 (with an AUC of 0.911 under the ROC curve), it can efficiently identify high-risk populations for thyroid dysfunction (with a sensitivity of 0.874 and specificity of 0.955), providing a reliable tool for early clinical intervention.

## Data Availability

The raw data supporting the conclusions of this article will be made available by the authors, without undue reservation.
