# Supplementary material for: Analysis of independent risk factors and construction of a predictive model for thyroid dysfunction in early pregnancy
Source: Front Endocrinol (Lausanne). 2025 Nov 14;16:1631445. doi: 10.3389/fendo.2025.1631445 (PMC12660063; doi:10.3389/fendo.2025.1631445)
Supplement: Supplementary file 1 [file Table1.docx]

Supplementary Material

**Table S1: Multicollinearity Diagnostics**

| Variables | VIF | Tolerance |
| --- | --- | --- |
| A-TPO | 1.079 | 0.927 |
| TSH | 1.078 | 0.927 |
| FT3 | 1.036 | 0.965 |

**Table S2: Results of the generalized linear mixed model for predictors of**

**thyroid dysfunction in early pregnancy**

| Fixed Effects | Coefficient (SE) | | Adjusted OR (95% CI) | | | *P* |  |
| --- | --- | --- | --- | --- | --- | --- | --- |
| (Intercept) | -8.55 (2.22) | |  | | | 0.0001 |  |
| A-TPO | 0.17 (0.07) | | 1.18 (1.03 - 1.36) | | | 0.021 |  |
| TSH | 6.42 (0.30) | | 613.74 (339.46 - 1109.64) | | | <0.001 |  |
| FT3 | 1.70 (0.18) | | 5.50 (3.84 - 7.88) | | | <0.001 |  |
| Random Effects | | | | | | |  |
| Groups | | Variance | | Standard Deviation | *P* | | |
| Center (Intercept) | | 13.18 | | 3.63 | 0.632 | | |

Model Specifications: Number of observations: 2151; Number of centers: 3; AIC: 1240.4. All continuous predictors were standardized (z-scores). OR: Odds Ratio; CI: Confidence Interval. P-value for the random effect was obtained by likelihood ratio test comparing the model with and without the random intercept.
